# Preface to the special issue on “Recent Advances in Functional Fibers”

**DOI:** 10.1007/s12200-022-00054-z

**Published:** 2022-12-29

**Authors:** Lei Wei, Guangming Tao, Chong Hou, Wei Yan

**Affiliations:** 1 School of Electrical and Electronic Engineering, Nanyang Technological University, Singapore, 639798 Singapore; 2 Wuhan National Laboratory for Optoelectronics and Sport and Health Initiative, Optical Valley Laboratory, Huazhong University of Science and Technology, Wuhan, 430074 China; 3 State Key Laboratory of Material Processing and Die and Mould Technology, School of Materials Science and Engineering, Huazhong University of Science and Technology, Wuhan, 430074 China; 4 School of Optical and Electronic Information, Huazhong University of Science and Technology, Wuhan, 430074 China; 5 School of Materials Science and Engineering, Nanyang Technological University, Singapore, 639798 Singapore

Fiber is one of the most fundamental material forms seen in human life. Befitting from their long and bendable shape, fibers with different specialties and different dimensions are used in a multitude of applications, ranging from fabrics [1] to telecommunications [2], from generating laser [3] to sensing and actuating, etc. [4–6]. In recent years, major breakthroughs were made, demonstrating that fibers have novel optical [7–9], electronic [10], acoustic [11, 12] and cell interfacing [13, 14] properties that enable new functionalities. Functional fibers and related application research are at the crossroads of many disciplines, including optics, materials science, device physics, nanotechnology, and fluid dynamics.

This special issue on “Recent Advances in Functional Fibers” is aimed to highlight the exciting developments in this field and to promote the diverse applications of functional fibers. The issue includes five review articles and three research articles, covering topics from theory and modelling of fiber to novel fiber manufacturing and applications in optical modulation, sensing, energy conversion, wearables, etc. Strutynski et al. [15] developed zinc-phosphate glasses doped with Nd_2_O_3_ of different concentrations, and observed linear evolution of the glass properties with respect to the Nd_2_O_3_ concentration. Based on that they produced multimode large rectangular-core fibers with a modified stack-and-draw method, and found that self-guided nonlinear effects acting as spatial beam reshaping processes in these newly-developed photonic structures led to the generation of spectral broadenings in the visible and near-infrared spectral domains. Ding et al. [16] proposed a novel all-solid anti-resonant single crystal fiber (AR-SCF) with high refractive index tubes as cladding and realized AR guiding, which can reduce the mode number to achieve single-mode or few-mode transmissions. They investigated the influences of different materials and structures on the confinement loss and effective guided mode number for wavelengths of 2–3 μm and determined the optimal AR-SCF structures for different wavelengths, and analyzed the influences of different fabrication errors. Li et al. [17] fabricated stretchable and color-changeable thermochromic fibers based on the wet spinning process. The fibers had good mechanical properties, excellent color change stability and could be prepared in large quantities. At the same time, the researchers also made fabrics with color-changing patterns, which could provide static and dynamic image display functions for information interaction, thus providing new possibilities for intelligent information interaction and information security protection.

Semiconductor optoelectronic fiber technology has seen rapid development in recent years thanks to advances in fabrication and post-processing techniques. Tsui et al. [18] summarized the recent progress of semiconductor optoelectronic fibers. They showed that a wide range of semiconductor materials had been demonstrated as amenable to fiber fabrication via the high pressure chemical vapor deposition (HPCVD), pressure assisted melt filling (PAMF), and molten core drawing (MCD) techniques, and semiconductor optoelectronic fibers not only extended the transmission range of optical fiber to the mid-infrared region but also broadened its uses in various applications, such as in-fiber frequency generation, signal modulation, photodetection, and multi-material functional fibers. It was anticipated that the semiconductor fiber platform would be important in a wide array of technological areas, from quantum optics, telecommunication, to wearable electronics. Zhang et al. [19] summarized the recent progress of fiber-based transistors, which is an important branch of semiconductor optoelectronic fibers, in terms of materials, structures and applications. The accessible materials for fiber-based transistors and their applications in sensing, memory, and logic computing are presented. They also discussed the current features, potentials, and challenges of fiber-based transistors in e-textile systems, and showed that fully integrated e-textile systems with high mechanical and environmental stability and minimum power consumption would become promising candidates for healthcare, sports, and entertainment applications.

Integrating functional materials and structures inside fibers enables new capabilities of fibers such as sensing, actuating, and energy conversion. Zhang et al. [20] provided an overview of the basic principles and applications of optical fiber magnetic field sensors. They discussed the working principle and structural features of different fiber configurations, such as fiber gratings (FBG, LPFG, and TFBG), fiber-based interferometry (MZI, FPI, SI, and MI), and tailored fiber with evanescent field (tapered fiber, D-shape fiber, and U-shape), as well as magnetically sensitive materials, including magnetic fluid materials, magnetic-strictive materials, and magneto-optical materials. They showed that high-precision distributed sensing systems of the magnetic field had important applications in geological monitoring and resource exploration, and the structured optical fibers and magnetically sensing materials could be combined to form integrated sensing fibers towards the high-precision distributed sensing system. Qian et al. [21] provided a comprehensive review of the thermally drawn piezoelectric fibers from aspects of materials, structures, fabrication, device integration and applications. They discussed the materials of piezoelectric fibers and the effect of fiber structure on the piezoelectric performance. It was shown that piezoelectric fibers could find applications in sensors for aquatic acoustic and audible sound, aids for communication and health care, detectors for space dust, underwater actuators, optical modulators, and energy harvesting. Chen et al. [22] introduced the fabrication process of nanofibers and the strategies to improve their performances and expand their functions. They showed that nanofibers could be fabricated via electrospinning, melt blowing, thermal drawing, or via chemical synthesis in templates. They reviewed various complex structures of nanofibers such as porous, Janus, core–shell, various material tuning methods such as blending polymer, doping catalysts, and different assembling styles of nanofibers, which would widely expand functionalities of nanofibers for applications in environmental sensing and treatment, energy generation and storage, as well as biomedical and health.

In the end, we hope that this special issue on “Recent Advances in Functional Fibers” could provide useful information for researchers working on fiber-related areas and inspire new ideas for their future exploration. We thank all authors for their contribution to this special issue, and reviewers for their valuable comments. We would like to express sincere gratitude to the editors of *Frontiers of Optoelectronics* for providing us such an excellent opportunity to put together this special issue and their invaluable assistance along the way.
